# The Robustness Analysis of Wireless Sensor Networks under Uncertain Interference

**DOI:** 10.1155/2013/185970

**Published:** 2013-11-30

**Authors:** Changjian Deng

**Affiliations:** ^1^School of Automation Engineering, University of Electronic Science and Technology of China, Chengdu 611731, China; ^2^Department of Control Engineering, Chengdu University of Information Technology, Chengdu 610225, China

## Abstract

Based on the complex network theory, robustness analysis of condition monitoring wireless sensor network under uncertain interference is present. In the evolution of the topology of sensor networks, the density weighted algebraic connectivity is taken into account, and the phenomenon of removing and repairing the link and node in the network is discussed. Numerical simulation is conducted to explore algebraic connectivity characteristics and network robustness performance. It is found that nodes density has the effect on algebraic connectivity distribution in the random graph model; high density nodes carry more connections, use more throughputs, and may be more unreliable. Moreover, the results show that, when network should be more error tolerant or robust by repairing nodes or adding new nodes, the network should be better clustered in median and high scale wireless sensor networks and be meshing topology in small scale networks.

## 1. Introduction

Currently, wireless sensor networks have been deployed for condition monitoring application. In industrial harsh environment, there are many kinds of uncertain interference, for example, energy dependence, dynamic topological update, and varying large number of nodes, and these make WSN a type of complex system.

Under uncertain industrial environment, robustness is an important property. Robustness is often defined as invariance degree of state, behavior, and function or the adaptation/flexibility degree under interference of perturbations. Robust analysis of wireless sensor networks is intractable and challenging.

There are three models of complex network [1–3]. The first model is the Erdős-Rényi model of random graphs, the second model is small-world model, and the third model is scale-free model of the power-law degree distribution.

Papers [4–12] proved that many complex systems display a surprising degree of tolerance for errors. Robustness of wireless complex networks can be enhanced by optimization of networks topology or by repair of its faults.

Papers [13–18] discuss that second smallest eigenvalue of the Laplacian matrix (algebraic connectivity) plays a special role for the robustness of networks using the Erdős-Rényi random graph, as an example for the model of condition monitoring wireless sensor networks.

Papers [19–27] study the ability to control networks. Recent work has extended the concept of pinning control and structural controllability to complex networks and so on.

With the fundamentals of these, the paper focuses on the research of topology choice and repairing control based on density weighted algebraic connectivity; when the vertex and links are not always constant, they can change with time.

The contributions of the paper are (1) weighted and changeable algebraic connectivity analysis in random network and (2) presenting a method to do topology choice and repairing control of different topology of wireless sensors, for example, the star topology, the tree-cluster topology, the mash topology, and so on.

The paper is arranged as follows: in Section 2, the related works are introduced; in Section 3, the research method of the paper is described; in Section 4, the simulation and its discussion are presented; in Section 5, the conclusion is given.

## 2. Related Work

### 2.1. Statistics Results of Erdős and Rényi Model

A network is represented as an undirected graph *G* = (*N*, *L*) consisting of *N* = |*N*| nodes and *L* = |*L*| links.

Erdős and Rényi define a random graph as *N* labeled nodes connected by *n* edges, which are chosen randomly from the *N*(*N* − 1)/2 possible edges. In total there are *C* ^*n*^_[*N*(*N*−1)/2]_ graphs with *N* nodes and *n* edges. An alternative and equivalent definition of a random graph is the binomial model, and following properties of the random graph can be determined asymptotically [1].

**Figure 1:**
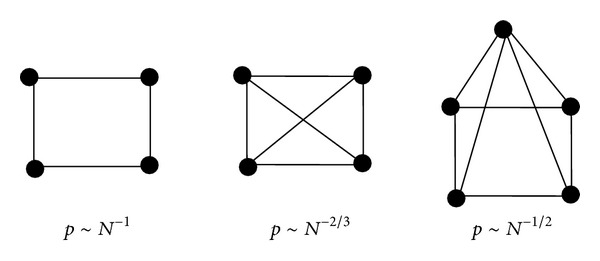
Threshold probabilities at which different subgraphs appear in a random graph.

(i) In ER model, there is a critical probability *p*_*c*_(*N*). If *p*(*N*) grows more slowly than *p*_*c*_(*N*) as *N* → *∞*, then almost every graph with connection probability *p*(*N*) fails to have property *Q*. If *p*(*N*) grows somewhat faster than *p*_*c*_(*N*), then almost every graph has the property *Q*.
(ii) The critical probability at which almost every graph contains a subgraph with *k* nodes and edges is *p*_*c*_(*N*) = *cN* ^−*k*/*l*^. (a) The critical probability of having a tree of order *k* is *p*_*c*_(*N*) = *cN* ^−*k*(*k*−1)^; (b) the critical probability of having a cycle of order *k* is *p*_*c*_(*N*) = *cN* ^−1^; (c) the critical probability of having a complete subgraph of order *k* is *p*_*c*_(*N*) = *cN* ^−2/(*k*−1)^.
(iii) The useful threshold probabilities for WSN at which different subgraphs appear in a random graph are shown in Figure 1. At *p* ~ *N* ^−1^ trees of all orders are present, and at the same time cycles of all orders appear. The probability *p* ~ *N* ^−2/3^ marks the appearance of complete subgraphs of order 4; *p* ~ *N* ^−1/2^ corresponds to complete subgraphs of order 5. As *z* approaches 0, the graph contains complete subgraphs of increasing order.
(iv) The expectation value of the number of nodes with degree *k* has a passion distribution.
(v) A general conclusion is that, for most values of *p*, almost all graphs with the same *N* and *p* have precisely the same diameter.
(vi) The clustering coefficient of a random graph is formula
  (1) $$\begin{array}{c} C_{\text{rand}}=p=\frac{\langle k\rangle }{N}. \end{array}$$

### 2.2. Algebraic Connectivity of Erdős and Rényi Model

The Laplacian matrix of a graph *G* with *N* nodes is an *N* × *N* matrix *Q* = Δ − *A*, where Δ = diag⁡ (*D*_*i*_). *D*_*i*_ denotes the nodal degree of the node *i* ∈ *N* and *A* is the adjacency matrix of *G*.

The asymptotic behavior of the algebraic connectivity in the Erdős-Rényi random graph *G*_*p*_(*N*) is as follows: for any *ε* > 0,

(2) $$\begin{array}{c} \lambda_{N-1}=pN+o\left(N^{1/2+\varepsilon }\right), \end{array}$$

where the algebraic connectivity converges in probability as *N* → *∞*.

Paper [18] defined that the correlation coefficient of the degrees *D*_*i*_ and *D*_*j*_ of two random nodes *i* and *j* in *G*_*p*_(*N*) for 0 < *p* < 1 is

(3) $$\begin{array}{c} \rho \left(D_{i},D_{j}\right)=\frac{\text{Cov}\left[D_{i},D_{j}\right]}{\sqrt{\mathrm{Var}\left[D_{i}\right]}\sqrt{\mathrm{Var}\left[D_{j}\right]}}=\frac{1}{N-1}. \end{array}$$

At large graph size *N*, the distribution of the algebraic connectivity will rapidly approach the mean value. And the distribution of the algebraic connectivity grows linearly with the minimum nodal degree, *λ*_*N*−1_ ≈ *D*_min⁡_.

And the larger the algebraic connectivity is, the better the graph's robustness of node and link failures is.

## 3. Weighted and Changeable Algebraic Connectivity of WSN

### 3.1. Weighted Algebraic Connectivity

**Figure 2:**
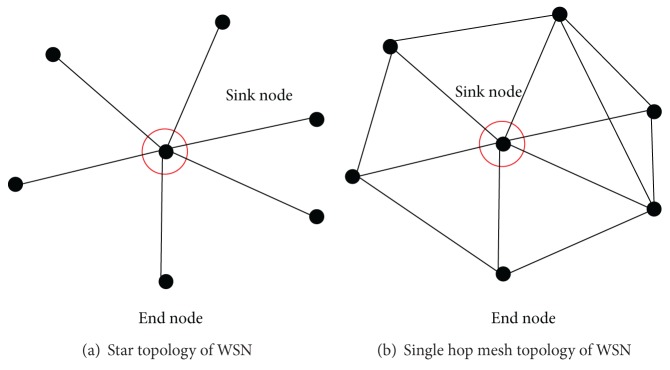
Simplest topology of normal condition monitoring WSN.

Figure 2 shows the simplest topology of normal equipment condition monitoring WSN.

In Figure 2, the vertex number of graph is seven. There is a very interesting phenomenon that if we omitted the vertex of sink node, the network should be almost connected with equal probability *p* (*k* of *N* nodes).

In time domain, this omitted network graph can be looked as a random graph. (For the data transmit, the connected link is at probability *p*.)

Here the average degree of a vertex in this network is

(4) $$\begin{array}{c} p_{k}=\left(\begin{array}{c} N-1\\ k \end{array}\right)p^{k}{\left(1-p\right)}^{N-1-k}≅\frac{z^{k}}{k!}e^{-z}. \end{array}$$

If *N* were large, this distribution should be looked to as the Poisson distribution.

**Figure 3:**
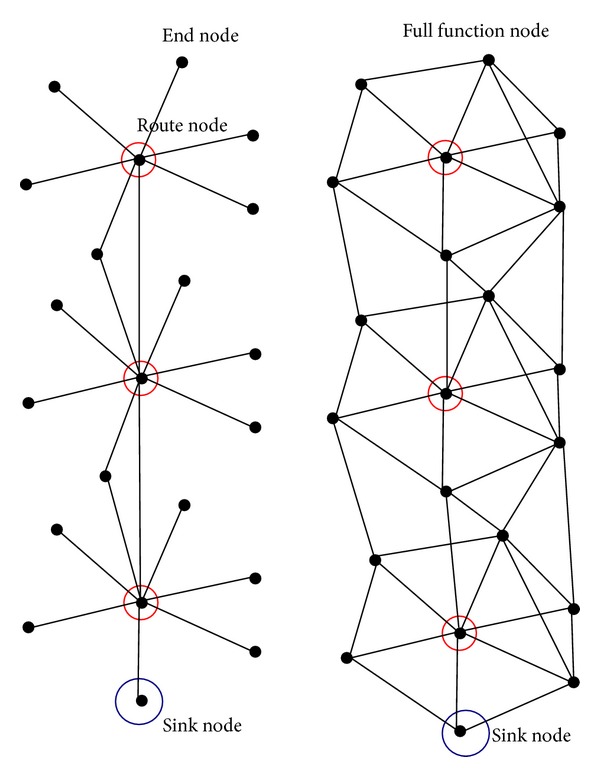
Modern complexity topology of normal condition monitoring WSN.

*(1) Medium Complexity Topology of Normal Condition Monitoring WSN.* As shown in Figure 3, the vertex number of graph is almost 21. In the left and right of Figure 3, the density of a network (number of nodes in an area) is the same, but possibility of links is different. There is also a phenomenon that if the vertex of route node was omitted, the network should be almost connected with equal probability *p* if they had the same density. And, if the density is large, the vertex degree of graph is large.

The omitted network graph can also be looked to as a random graph. And the network made from route nodes and sink nodes has similar property with mesh network.

*(2) Large Complexity Topology of Condition Monitoring WSN*. When there are hundreds of nodes in a wireless condition monitoring network and the network consists of many similar areas that have independent functions, and then we can define this every area as a cell of network. Then if the cell of network has the same topology as Figure 2, then it has the similar results as (1).

The eigenvalues of *Q* are called the Laplacian eigenvalues. The Laplacian eigenvalues *λ*_*N*_ = 0 ⩽ *λ*_*N*−1_ ⩽ ⋯⩽*λ*_1_ are all real and nonnegative. The second smallest Laplacian eigenvalue *λ*_*N*−1_ is also known as the algebraic connectivity.

**Lemma 1**

Algebraic connectivity of wireless star, cluster-tree, or mesh network has below properties:

 For an ideal full-function mesh network, algebraic connectivity is proportional with density of network.
 For an ideal star or cluster-tree network, the end nodes have similar algebraic connectivity; algebraic connectivity of the route nodes and AP (or coordinator) is proportional with density of network.
 For complex WSN, algebraic connectivity has a weight; the simplest example of the weight is density of network.

### 3.2. The Influence Function of Weighted Algebraic Connectivity

To discuss the robustness of WSN, here two connectivity metrics of *G* are introduced: (1) the link (edge) connectivity *κ*_*L*_ is the minimal number of links whose removal would disconnect *G*; (2) the node (vertex) connectivity *κ*_*N*_ is defined analogously (nodes together with adjacent links are removed).

The robustness of network graph has a relationship with algebraic connectivity; the algebraic connectivity of a graph is increased with the node and the link connectivity. In another way, robustness has a relationship with error tolerance of network. Two types of node removal were considered. The first type was that all the nodes were randomly removed. The second type was that the most highly connected nodes were of more reliability nodes; other was the same as type 1.

**Definition 2 (influence function set of network)**

In graph *R* of network, if the subgraph *R*_1_ is not connected with Sink (or network access point node) node, then subgraph is *R*_1_ graph, and connected graph is *R*-*R*_1_ graph.

If *R*_1_ occurred from removal of node *k*, then ratio of *E*_*k*_ = *R*_1_/*R* means the destroy effect of node *k*, called influence function of node *k*.

The sum of *E*_*i*_ is called one-dimension influence function of network

(5) $$\begin{array}{c} \sum_{i=1}^{n}E_{i}=\text{I}{\text{F}}_{\text{net}},\;i\in R. \end{array}$$

If *k*_1_, *k*_2_ were removed, *E*_1,2_ were called influence function of nodes *k*_1_, *k*_2_. If the removed nodes were sets, for example, the removed nodes sets are *℘K* = {*K*_1_, *K*_2_,…}, *K*_1_ = {*k*_1_, *k*_2_}, *K*_2_ = {*k*_3_, *k*_4_}, then the *℘E* = {*E*_*K*_1__, *E*_*K*_2__,…} is the influence function set of network.

**Definition 3 (influence function set of weighted algebraic connectivity)**

Propose the density (weighted) stands for more easy to produce congest, and to be more unreliability. Here density weighted influence function set of network is defined. It has a relationship with throughput, energy cost, and so on.

**Lemma 4**

An ideal random network means that there are no determining factors that can infect network communication, for example, (nodes) density, (effective) communication distances. Then the mean or least square mean of connectivity is optimum estimation value, and meanwhile the network may be microstable (less intermoving) or reliable.

*Note*. In ZigBee, there are phenomena of near neighbor ring, hidden nodes, and so on. In WirelessHart, there are phenomena of limited connective number, loss of noisy node, and so on. They have not been studied in detail in the paper.

### 3.3. Changeable Algebraic Connectivity

There are some different kinds of model of time-varying topologies: (1) the switching topology which refers to a deterministically time-varying model where, at each time instant, the network adopts a topology from a known set; (2) random topologies, in the presence of random failures caused by working under uncertain interference, for instance, changes in the environment, mobility of the nodes, asynchronous sleeping periods, or randomized communication protocols; the topology of a WSN varies randomly with time; (3) E-R model.

When wireless sensor networks are working under uncertain interference, nodes and links may lose effect momentarily or permanently. And the topology may be different with random topologies.

When the links are added or removed unpredictably from the set at any time, the graph can be looked to as the realization of a random process. WSN are normally exposed to random communication failures caused by uncertain interference, and these communication impairments cause abrupt changes in the connectivity of the network, which are described by means of a random graph,

(6) $$\begin{array}{c} A_{k}=A_{k-1}\cdot B_{t},\\ B_{t}=I,\;t<t_{c},\\ B_{t}=I+\Delta B,\;t>t_{c}. \end{array}$$

When considering formula (6), the connectivity matrix *A* is changed after time *t*_*c*_. And meanwhile the algebraic connectivity of graph is changed.

Then the algebraic connectivity *λ*_*N*−1_ is random variable; its distribution with the losing effective nodes is important to improve the robustness of complex networks, by optimization of complex networks topology or by repairing the damage nodes of complex networks.

## 4. Simulation and Test Results

The state of the network can be simplified as linear system:

(7) $$\begin{array}{c} \dot{x}\left(t\right)=-Lx\left(t\right), \end{array}$$

where *x*(*t*) = [*x*_1_(*t*), *x*_2_(*t*),…, *x*_*N*_(*t*)]^*T*^ is the vector of all states at time *t* and **L** ∈ *ℜ* ^*N*×*N*^ is the Laplacian matrix associated with the graph.

A discrete implementation of the expression of (7) is

(8) $$\begin{array}{c} x_{i}\left(k+1\right)=\sum_{j\in N_{i}\cup \{i\}}{w_{i}}_{j}x_{j}\left(k\right),\;k\geq 0. \end{array}$$

Different from weighted algebraic connectivity, *w*_*ij*_ is nonzero weight assigned by node *i* to the information received from node *j*, satisfying

(9) $$\begin{array}{c} \sum_{j\in N_{i}\cup \{i\}}w_{ij}=1. \end{array}$$

More generally, the linear control systems are described by the following state equation:

(10) $$\begin{array}{c} \dot{x}\left(t\right)=Ax\left(t\right)+Bu\left(t\right), \end{array}$$

where *x*(*t*) = {*x*_1_(*t*), *x*_2_(*t*),…*x*_*N*_(*t*)}^*T*^, which is the state of a system of *N* nodes at time *t*. *A* is the *N* × *N* adjacency matrix of the network representing the system. *B* is the *N* × *M* input matrix, which identifies the nodes where the input signals are imposed. The input signal vector *u*(*t*) = {*u*_1_(*t*), *u*_2_(*t*),…*u*_*N*_(*t*)}^*T*^ is a time-dependent input signal vector. The state of each node at each time step is controlled by the linear combination of the elements of the input vector.

### 4.1. Weighted Algebraic Connectivity and Not Weighted Algebraic Connectivity

**Figure 4:**
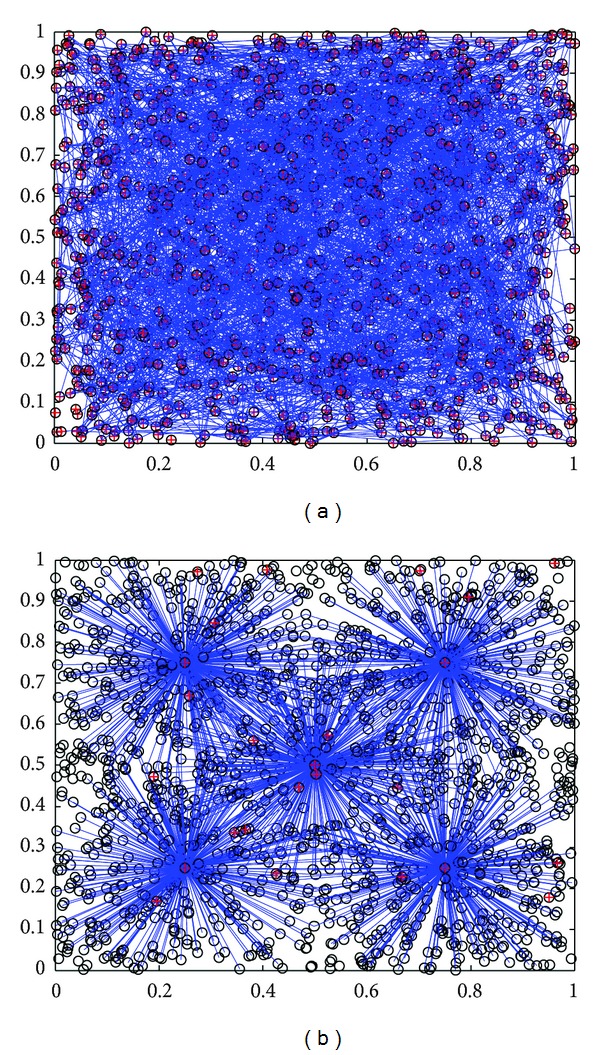
Different topology and its communication links in a certain area (left is random mesh; right is star).

In wireless condition monitoring network, supposing whether nodes can be connected or not only relies on its effective communication distance. As shown in Figure 4, the density of nodes and the topology of networks are two important factors to research communication links.

*(1) The Relationship between Density and Connectivity.* Simulate method: in certain area, using different numbers of nodes circulate its connectivity.

**Figure 5:**
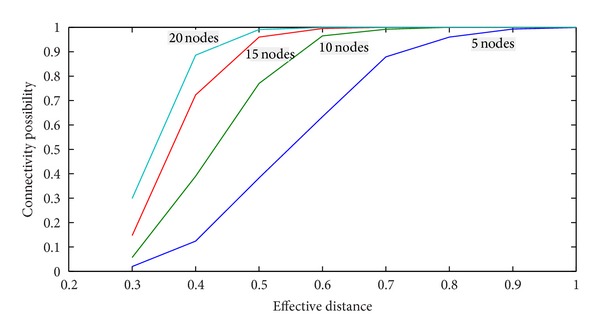
The relationship between density and connectivity.

The connectivity is defined as the successful connection possibility when random deploy nodes in this area 100 times. When numbers of nodes are above than or equal to 10, the networks have a reliability of connectivity (as shown in Figure 5). So the theory of effective distance is less than 0.47.

This means, in wireless condition monitoring network, an effective distance of random network should be larger than half of monitoring area. Considering its physical communication ability, the edge of monitoring cell is defined as twice times of its effective distance.

**Figure 6:**
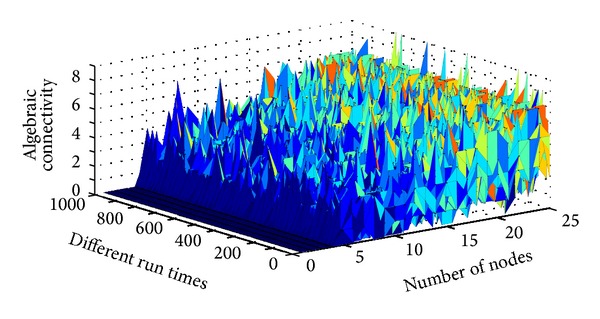
The relationship between algebraic connectivity and connectivity.

*(2) The Relationship between Algebraic Connectivity and Density When the Effective Distance Is about 0.5.* As shown in Figure 6, discrete algebraic connectivity is varied with the density (or number of nodes in certain area); the useful value of algebraic connectivity is a flat plain of certain density (10–20 nodes in a cell). The reason is too large the density form another different subgraphs and outlier of algebraic connectivity. 

**Figure 7:**
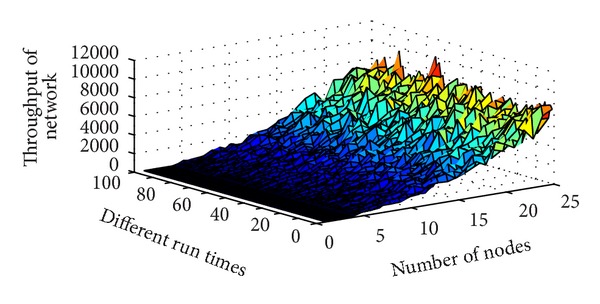
The relationship between throughput of network and density.

*(3) The Relationship between Network Throughput and Density*. In simulation, throughput is only comprised of the transmitting sense data; the network management steam is not considered. As shown in Figure 7, when density of network is large, the throughput of network became very large, such that the value is almost 10 k byte per second. So the density of network should not be large.

As discussed in (2) and (3), this gives the property of density weighted algebraic connectivity.

### 4.2. Robustness Analysis of Random Complex Network

**Figure 8:**
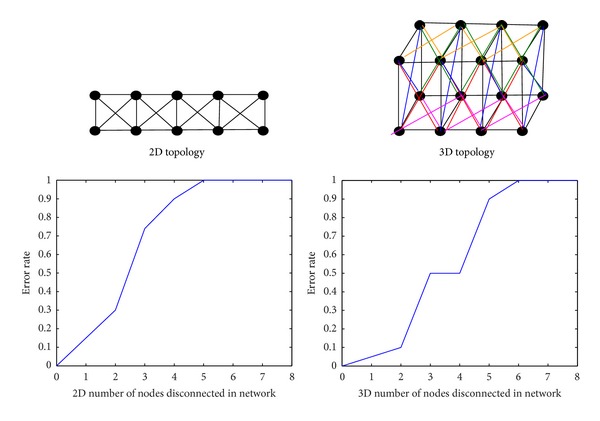
The relationship between random destruction number of node and connectivity.

*(1) The Relationship between Random Destruction Number of Node and Connectivity*. As shown in Figure 8, when random destruction of node is larger than its degree, then the connection of network can be destroyed.

*(2) The Robustness Analysis of Random Complex Network*. Consider a new graph that includes the plant's sensors and actuator, where the plant is controlled using a multihop wireless network. In applied layer application, condition monitoring parameters are measured by sensors and transmitted in networks; the networks construction changed with uncertain interference; this output is different with ordinary output with its random delay, packets loss rate, and so on.

There are two kinds of data; one kind is distributed data. It combined lots of nodes to get a useful data, as shown in formula

(11) $$\begin{array}{c} x\left[k+1\right]=\left(A+\Delta A\right)x\left[k\right]+\left(B+\Delta B\right)u\left[k\right],\\ y\left[k\right]=\left(C+\Delta C\right)x\left[k\right]. \end{array}$$

The another kind is standalone data, when can be described as a function, like

(12) $$\begin{array}{c} y_{i}\left(t\right)=f\left(x_{i}\left(t\right)\right)+\Delta f\left(x_{i}\left(t\right)\right). \end{array}$$

An example delay of wireless HART network is

(13) $$\begin{array}{c} T\_d=-0.0011\times n^{2}+0.24\times n-1.3333,\;n>50,\\ T\_d=0.1029\times n+2,\;n<50. \end{array}$$

Suppose that loss of effective nodes is relay nodes, and then delay time value is changed in normality distribution way; its means is zero. Its standard deviation has a relationship with connectivity.

**Figure 9:**
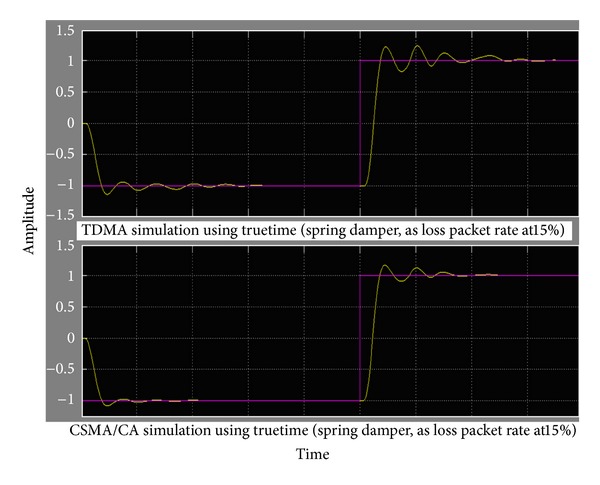
The relationship between loss packet rate and step response of spring damper.

As a result, if delay time is shorter than sample time, delays will do less interference with LTI system. And if the packet loss rate was less than 15% (use true time simulate using CSMA/CA, TDMA), spring damper system should work normally with little performance degrade (as shown in Figure 9).

If the system is not connected, should repair it. This robust control problem is to find control scheme to obtain system robustness. That includes two factors: the first is topology selection; the second is repairing scheme.

**Assumption 5**

If the connected degree distribution was Poisson distribution, the Packet loss rate under uncertain interface should follow the Poisson distribution.

Then this becomes a time-space transmitting processing problem.

**Figure 10:**
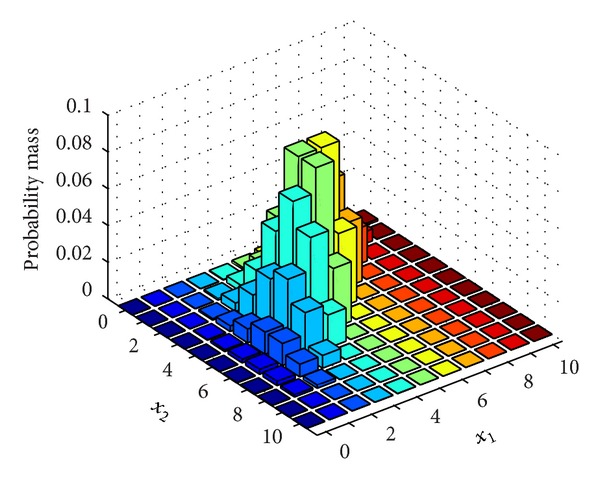
The time and space domain losing packet rate at lower space density.

**Figure 11:**
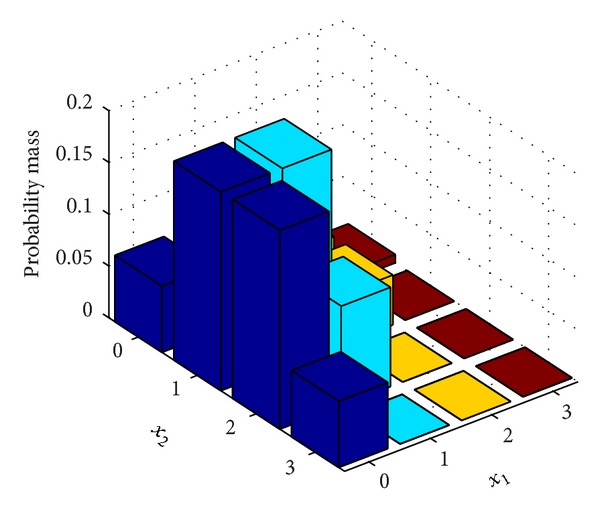
The time and space domain losing packet rate at higher space density.

In every time slot different density (data transmitting rate), the most possibility of losing packet is shown in Figures 10 and 11.

*Conclusion*. It is obvious that the cluster-tree, star-mesh topologies are easier than only mesh network to repair the network for its small algebraic connectivity and communication links. And it uses self-repairing or deploying new nodes to repair network.

But center nodes are more important and fragile than other nodes in these topologies. If they are to be novel stronger than other nodes, in median and large networks, this topology may have large possibility to be more robust than mesh topology in median and large scale networks. And, in small scale network, mesh topology may have large possibility to be more robust than other topology.

## 5. Conclusions

The cluster-tree, star-mesh topologies are easier than only mesh network to repair the network and may have large possibility to be more robust than mesh topology in median and large scale networks. In small scale network, mesh topology may have large possibility to be more robust than other topology.

If random destruction of node was larger than its algebraic connectivity, the connection of network should be broken out.

The properties of density weighted algebraic connectivity are two factors: one is that the useful value of algebraic connectivity is in certain density; the other is an effective network in which the density of network should not be large.
